# *In vivo* inactivation of glycosidases by conduritol B epoxide and cyclophellitol as revealed by activity‐based protein profiling

**DOI:** 10.1111/febs.14744

**Published:** 2019-02-02

**Authors:** Chi‐Lin Kuo, Wouter W. Kallemeijn, Lindsey T. Lelieveld, Mina Mirzaian, Iris Zoutendijk, Ayelet Vardi, Anthony H. Futerman, Annemarie H. Meijer, Herman P. Spaink, Herman S. Overkleeft, Johannes M.F.G. Aerts, Marta Artola

**Affiliations:** ^1^ Department of Medical Biochemistry Leiden Institute of Chemistry Leiden University The Netherlands; ^2^ Department of Biomolecular Sciences Weizmann Institute of Science Rehovot Israel; ^3^ Institute of Biology Leiden University The Netherlands; ^4^ Department of Bio‐organic Synthesis Leiden Institute of Chemistry Leiden University The Netherlands; ^5^ Present address: Department of Chemistry Imperial College London London UK

**Keywords:** activity‐based probes, conduritol B epoxide, cyclophellitol, Gaucher disease, glucocerebrosidase

## Abstract

Glucocerebrosidase (GBA) is a lysosomal β‐glucosidase‐degrading glucosylceramide. Its deficiency causes Gaucher disease (GD), a common lysosomal storage disorder. Carrying a genetic abnormality in GBA constitutes at present the largest genetic risk factor for Parkinson's disease (PD). Conduritol B epoxide (CBE), a mechanism‐based irreversible inhibitor of GBA, is used to generate cell and animal models for investigations on GD and PD. However, CBE may have additional glycosidase targets besides GBA. Here, we present the first *in vivo* target engagement study for CBE, employing a suite of activity‐based probes to visualize catalytic pocket occupancy of candidate off‐target glycosidases. Only at significantly higher CBE concentrations, nonlysosomal glucosylceramidase (GBA2) and lysosomal α‐glucosidase were identified as major off‐targets in cells and zebrafish larvae. A tight, but acceptable window for selective inhibition of GBA in the brain of mice was observed. On the other hand, cyclophellitol, a closer glucose mimic, was found to inactivate with equal affinity GBA and GBA2 and therefore is not suitable to generate genuine GD‐like models.

**Enzymes:**

Glucocerebrosidase (http://www.chem.qmul.ac.uk/iubmb/enzyme/EC3/2/1/45.html), nonlysosomal β‐glucocerebrosidase (http://www.chem.qmul.ac.uk/iubmb/enzyme/EC3/2/1/45.html); cytosolic β‐glucosidase (http://www.chem.qmul.ac.uk/iubmb/enzyme/EC3/2/1/21.html); α‐glucosidases (http://www.chem.qmul.ac.uk/iubmb/enzyme/EC3/2/1/20.html); β‐glucuronidase (http://www.chem.qmul.ac.uk/iubmb/enzyme/EC3/2/1/31.html).

## Graphical abstract

*In vivo* target engagement of mechanism‐based glucocerebrosidase (GBA) inhibitors—conduritol B epoxide (CBE) and cyclophellitol (CP)—were examined in cultured cells, zebrafish larvae and mice by competitive activity‐based protein profiling (ABPP). This method utilizes suicide fluorescent enzyme reporter molecules to assess active site occupancy of target glycosidases by inhibitors. The *in vivo* targets of CBE and CP and their selectivity towards GBA were revealed.

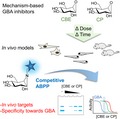

**Abbreviations**
ABP: activity‐based probe
ABPP: activity‐based protein profiling
CBE: conduritol B epoxide
CP: cyclophellitol
dpf: days postfertilization
GBA: glucocerebrosidase
GD: Gaucher disease
GlcSph: glucosylsphingosine
PD: Parkinson's disease

## Introduction

The lysosomal enzyme glucocerebrosidase (GBA, http://www.chem.qmul.ac.uk/iubmb/enzyme/EC3/2/1/45.html) is a retaining β‐glucosidase that degrades the glycosphingolipid, glucosylceramide. Inherited deficiencyof GBA is the cause of autosomal recessive Gaucher disease (GD) 1. Most GD patients display heterogeneous symptoms including spleen and liver enlargement, bone deterioration, anaemia, leukopenia and thrombocytopaenia. Some patients also develop fatal neurological symptoms 2. The GBA genotype poorly predicts the onset and severity of disease in individual GD patients, even in monozygotic twins 3, 4. Carriers of a GBA defect do not develop GD but show a markedly increased risk for Parkinson's disease (PD) and Lewy body dementia 5, 6. The molecular basis for this risk is unknown and a subject of research.

**Figure 1:**
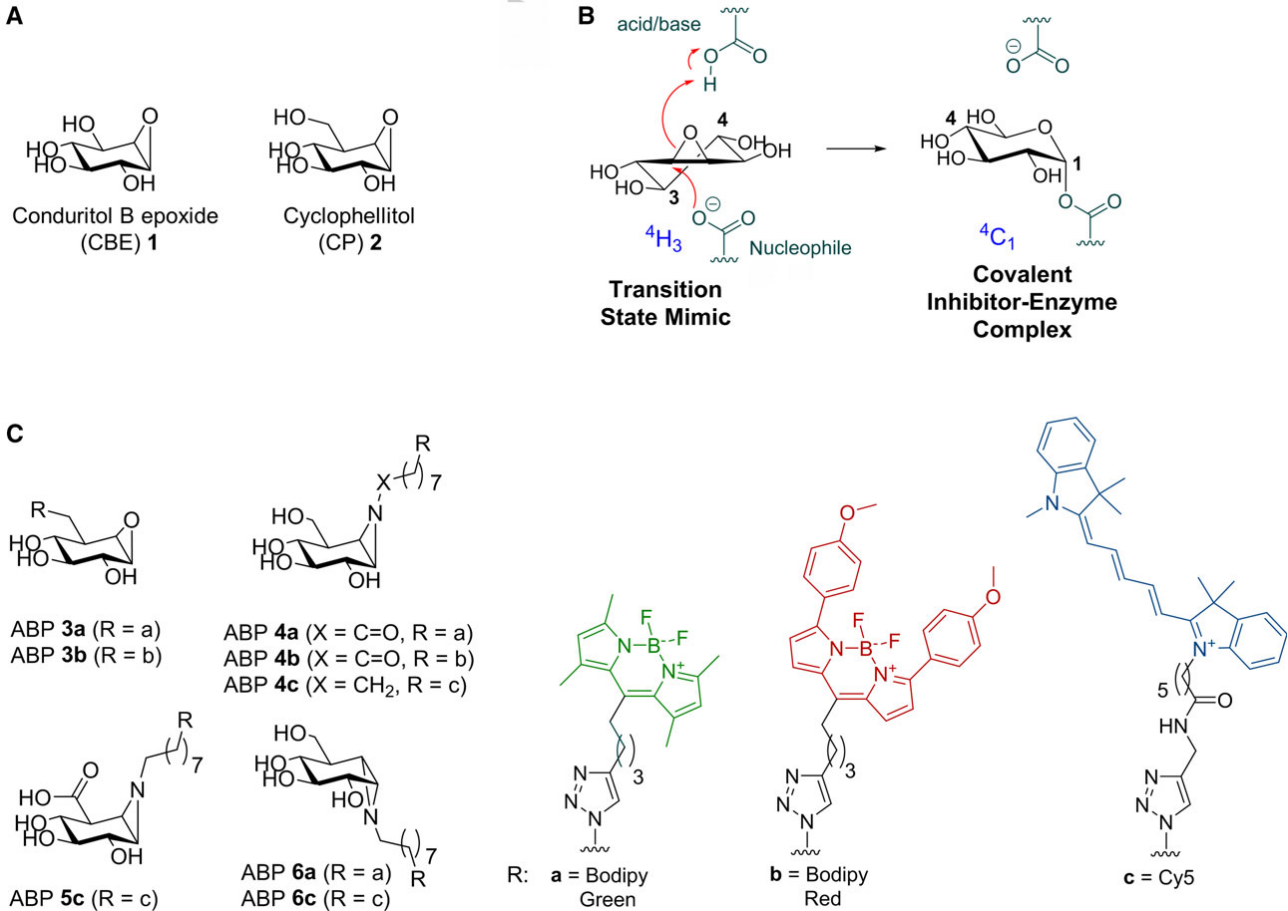
Structures of compounds used in this study and inactivation of β‐glucosidases by CBE. (A) Chemical structure of CBE **1** and cyclophellitol (CP) **2**. (B) Reaction coordinates of CBE during inhibition of β‐glucosidases. (C) Activity‐based probes (ABPs) used in this study: GBA ABPs **3a** and **3b**, GBA and GBA2 ABPs **4a**‐**c**, GUSB ABP **5c**, and GAA and GANAB ABPs **6a** and **6c**.

Because complete genetic abrogation of GBA results in premature death in mice, research models of GBA deficiency are often generated with conduritol B epoxide (CBE) (Fig. 1A) 7, 8, 9. CBE is a cyclitol epoxide that covalently and irreversibly reacts with the catalytic nucleophile of GBA and thus inactivates irreversibly the enzyme (Fig. 1B). The crystal structure of GBA with bound CBE confirmed the covalent linkage of the compound to the catalytic nucleophile Glu340 10, 11. Building on the initial work by Kanfer and coworkers, a regimen using different doses of CBE has been established to generate a phenotypic copy of neuronopathic GD in mice 9, 10, 11, 12. This pharmacological model is now widely used to study the nature of neuropathology resulting from GBA deficiency, including Parkinsonism 13, 14, 15.

A major advantage of CBE's pharmacological use in cultured cells and mice is its tunability: the extent of GBA inactivation can be adjusted by variation in the inhibitor concentration and/or exposure time 9. However, this has led to the use of distinct treatment regimens across studies: exposure of cells ranging from 50 μm to 100 mm CBE for 2 h up to 60 days 16, 17, 18, 19, 20, 21, 22 and daily exposure of mice from 25 to 300 mg·kg^−1^ body weight during 2 h up to 36 days 9. The use of a high CBE concentration raises concerns about specificity since the compound has been reported to inhibit at high concentration other glycosidases than GBA. Examples are *in vitro* inhibition of retaining α‐glucosidases (http://www.chem.qmul.ac.uk/iubmb/enzyme/EC3/2/1/20.html) 23, 24, 25, 26, *in vitro* 27 and *in situ* 28 cell inhibition of the nonlysosomal glucosylceramidase (GBA2, http://www.chem.qmul.ac.uk/iubmb/enzyme/EC3/2/1/45.html), and inhibition of the lysosomal β‐glucuronidase (GUSB, http://www.chem.qmul.ac.uk/iubmb/enzyme/EC3/2/1/31.html) in mice 29. The reactivity of CBE towards both β‐ and α‐glucosidases can be explained by the C2 symmetry found in its structure 26 (Fig. 1B), which allows reaction with the catalytic nucleophile of both classes of enzymes. Another structurally related cyclitol epoxide, cyclophellitol (CP, Fig. 1A), is a structurally closer β‐glucose mimic and inhibits GBA with far higher affinity than CBE 30, 31. It exhibits selectivity over α‐glucosidases due to the C5‐hydroxymethyl group 30, 31, 32, and was also shown to induce Gaucher phenotypes in mice 30. Its reactivity *in vivo* towards GBA2 and other glycosidases is unknown.

Our aim was to systematically study the *in vivo* selectivity of CBE and CP in cells and animal models. We envisioned that besides the traditional enzymatic assays employing fluorogenic substrates, activity‐based probes (ABPs) could be superior tools for this study. Unlike enzymatic substrate assays, which do not easily distinguish similar enzymatic activities such as GBA vs GBA2, ABPs would allow direct and unambiguous visualization of respective target glycosidases that are not occupied/inactivated by CBE or CP at the active site pocket. Cravatt and coworkers and van der Stelt and colleagues earlier used ABPs directed towards proteases and lipases in a competitive activity‐based protein profiling (ABPP) approach to identify *in vivo* target engagement of small compounds 33, 34, 35. For our study, we used cyclophellitol‐epoxide ABP tagged with a fluorophore that labels specifically GBA 32, and appropriately configured cyclophellitol‐aziridines tagged with a fluorophore that label multiple β‐glucosidases, (GBA and GBA2) 36, β‐glucuronidase (GUSB) 37 and α‐glucosidases (GAA and GANAB) 38 (Fig. 1C, ABPs **3**–**6**). Here, we report a detailed *in vivo* target engagement study for CBE and CP. Through parallel application of both the competitive ABPP method and enzymatic assay in lysates of cultured cells, zebrafish (*Danio rerio*) larvae, and brains of mice treated *in vivo* with a relevant range of concentrations of CBE or CP, we have systematically assessed their *in vivo* off‐targets and selectivity windows for GBA.

## Results

### A competitive ABPP method to determine GBA target engagement of CBE and CP

**Figure 2:**
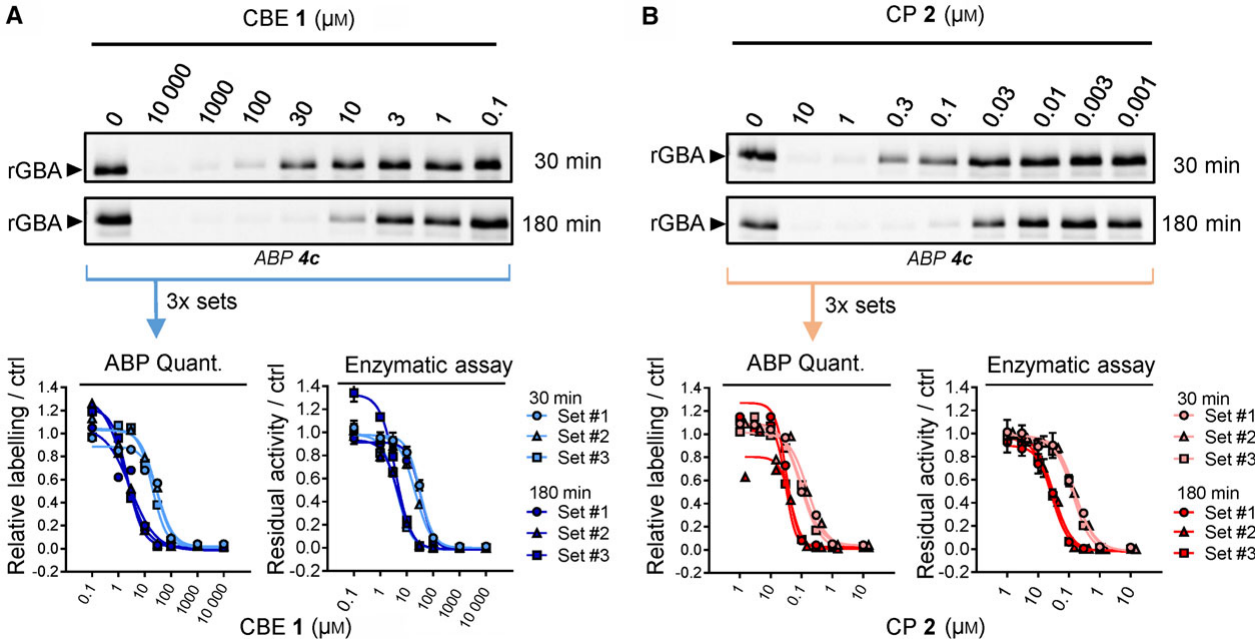
Effect of preincubation with CBE or CP on ABP labelling of recombinant GBA. (A) Representative gel images (1 set from *n *= 3 biological replicates) of rGBA preincubated with CBE for 30 min or 180 min and fluorescently labelled with ABP **4c**. Fluorescent signals were quantified and normalized to the untreated sample (ctrl, 0 μm CBE) (bottom left), and compared to the inhibition curves obtained with enzymatic assay (bottom right). **(**B**)** Same as A, with CP at 10–0.001 μm. Error ranges in enzymatic assay = ±SD,* n *= 3 (technical replicates).

We previously generated ABPs specific towards GBA and demonstrated their use in profiling GBA in cells and identifying active site pocket interactors (Fig. 1C, ABP **3** and **4**) 32, 39. Here, we examined their value to visualize GBA target engagement of CBE **1** and CP **2** with a competitive ABPP method by which the irreversible occupancy of the catalytic nucleophile of GBA by the inhibitors during preincubation is assessed. As a validation, competition of ABP labelling by CBE and CP was compared to the loss of GBA activity measured using 3.75 mm 4‐methylumbelliferyl‐β‐glucoside as substrate 40. For this, recombinant human GBA (rGBA) was preincubated with CBE across a range of concentrations at 37 °C for 0, 30 and 180 min in McIlvaine buffer (pH 5.2) containing 0.2% taurocholate and 0.1% Triton X‐100, a condition optimal for enzymatic activity 41. Subsequent labelling of GBA by ABP **4c** was quantified by SDS/PAGE and fluorescence scanning. IC_50_ values (concentrations of inhibitor yielding a 50% reduction of ABP **4c** labelling) were determined and found to be 26.6 μm at 30 min CBE preincubation, and 2.30 μm at 180 min preincubation (**Fig.** 2 **A**, Table**** 1). These values match the ones determined by the measurement of residual enzymatic activity of GBA assay (Fig.**** 2A lower right panel, Table**** 1), validating the competitive ABPP methodology.

**Table 1:** *In vitro* inhibition of CBE or CP towards human recombinant GBA. Apparent IC_50_ values (μm) were derived from the average of three individual experiments as measured by either enzymatic assays (*Enz. assay*) or quantification of ABP labelling on residual active enzymes (*ABP*). Error ranges = ±SD, *n *= 3 biological replicates

Incubation time	CBE		CP	
	Enz. assay	ABP	Enz. assay	ABP
30 min	28.8 (±6.90)	26.6 (±7.03)	0.150 (±0.013)	0.118 (±0.034)
180 min	4.28 (±0.500)	2.30 (±0.580)	0.030 (±0.002)	0.036 (±0.008)

Next, CP was comparably studied and its IC_50_ values determined by ABPP were 0.15 μm at 30 min preincubation and 0.03 μm at 180 min preincubation, comparable to values determined by enzymatic assay (Fig. 2B, Table 1).

### *In vivo* targets of CBE

**Figure 3:**
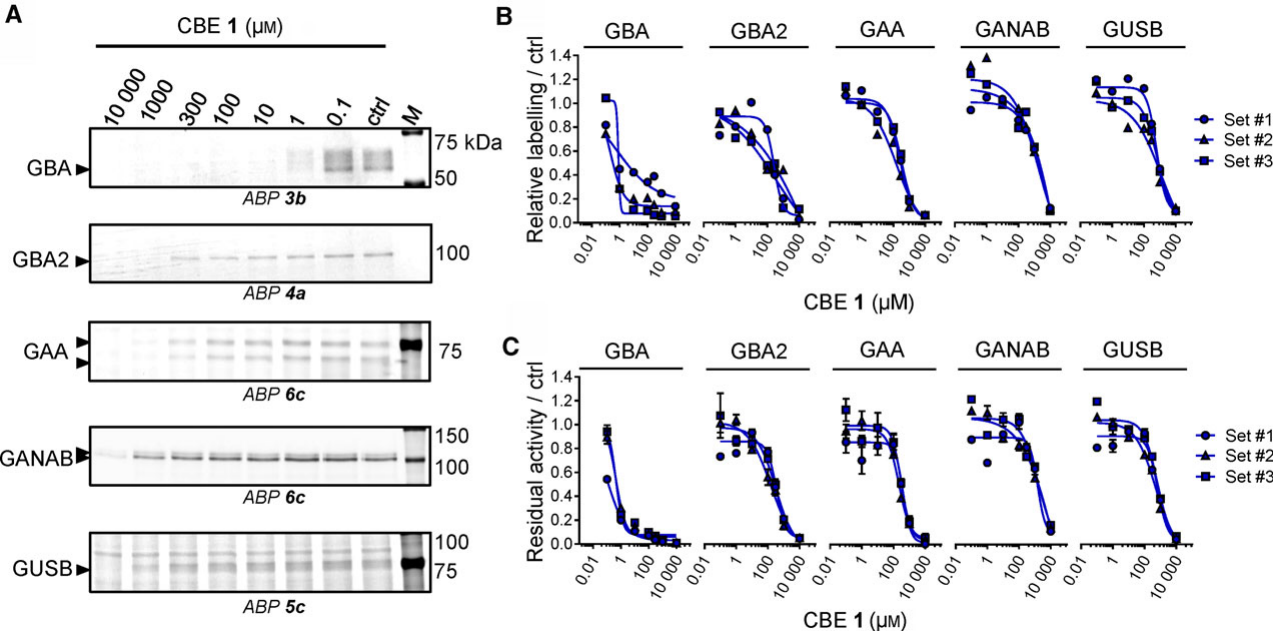
*In vivo* glycosidase targets of CBE in cultured cells. (A) Representative gel images (1 set from *n *= 3 biological replicates) showing fluorescent ABP labelling of GBA, GBA2, GAA, GANAB and GUSB in lysates of cells treated *in vivo* for 24 h with CBE. **(**B) Quantification of relative ABP labelling. (C) Residual activities of glycosidases in cell lysates treated *in vivo* with CBE. Error ranges = ±SD,* n *= 3 (technical replicates).

**Figure 4:**
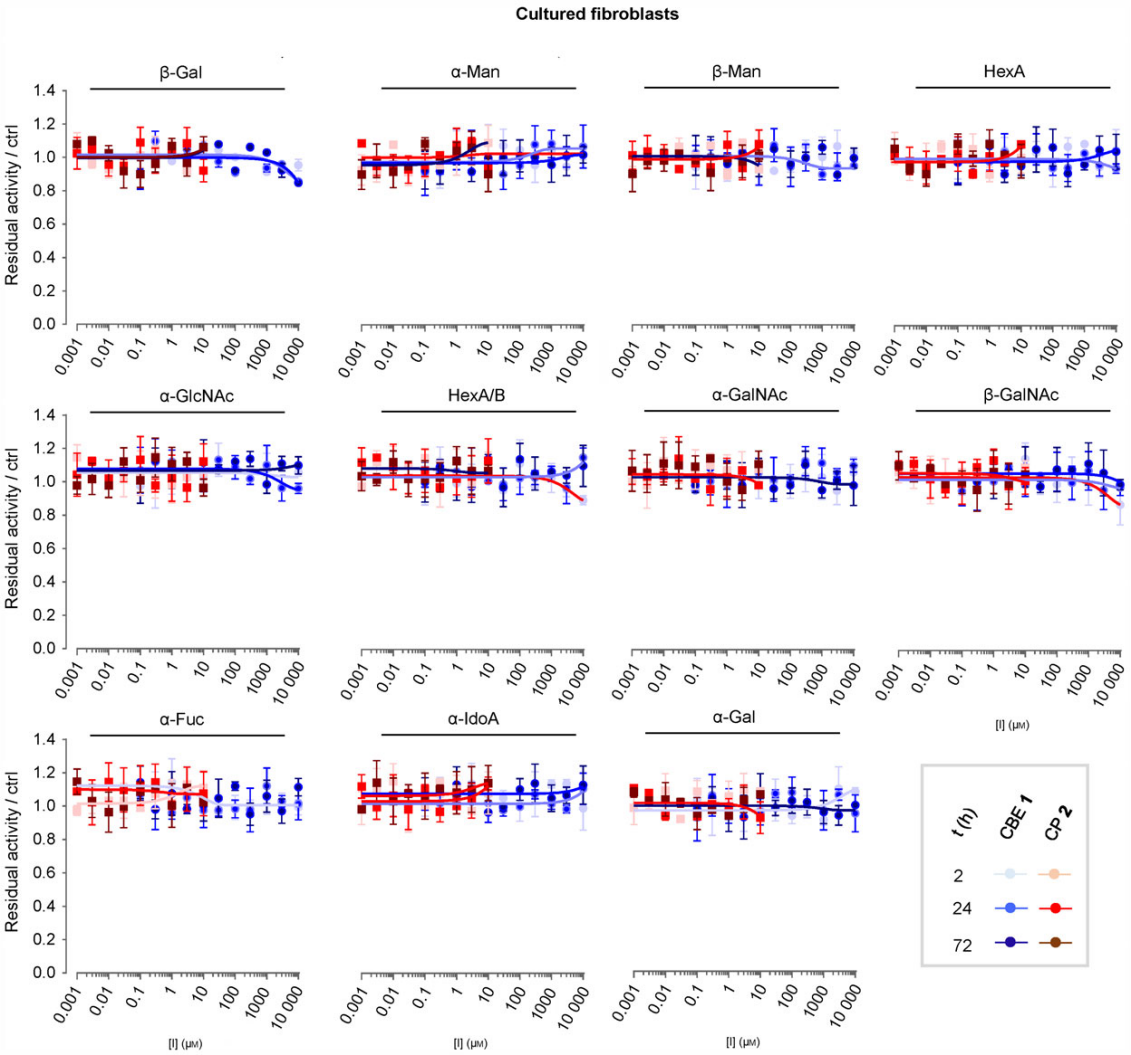
Effect of *in vivo* incubation with CBE and CP on enzymatic activity of glycosidases in cultured cells. Confluent cultured human fibroblasts were treated *in vivo* with different concentrations of CBE (blue) and CP (red) for 2 h, 24 h or 72 h, before being harvested and lysed in potassium phosphate buffer. Residual activities of glycosidases were measured by *in vitro* enzymatic assay using appropriate glycoside substrates, and normalized to the activity from the control samples. Error range = ±SD,* n *= 3 technical replicates.

**Figure 5:**
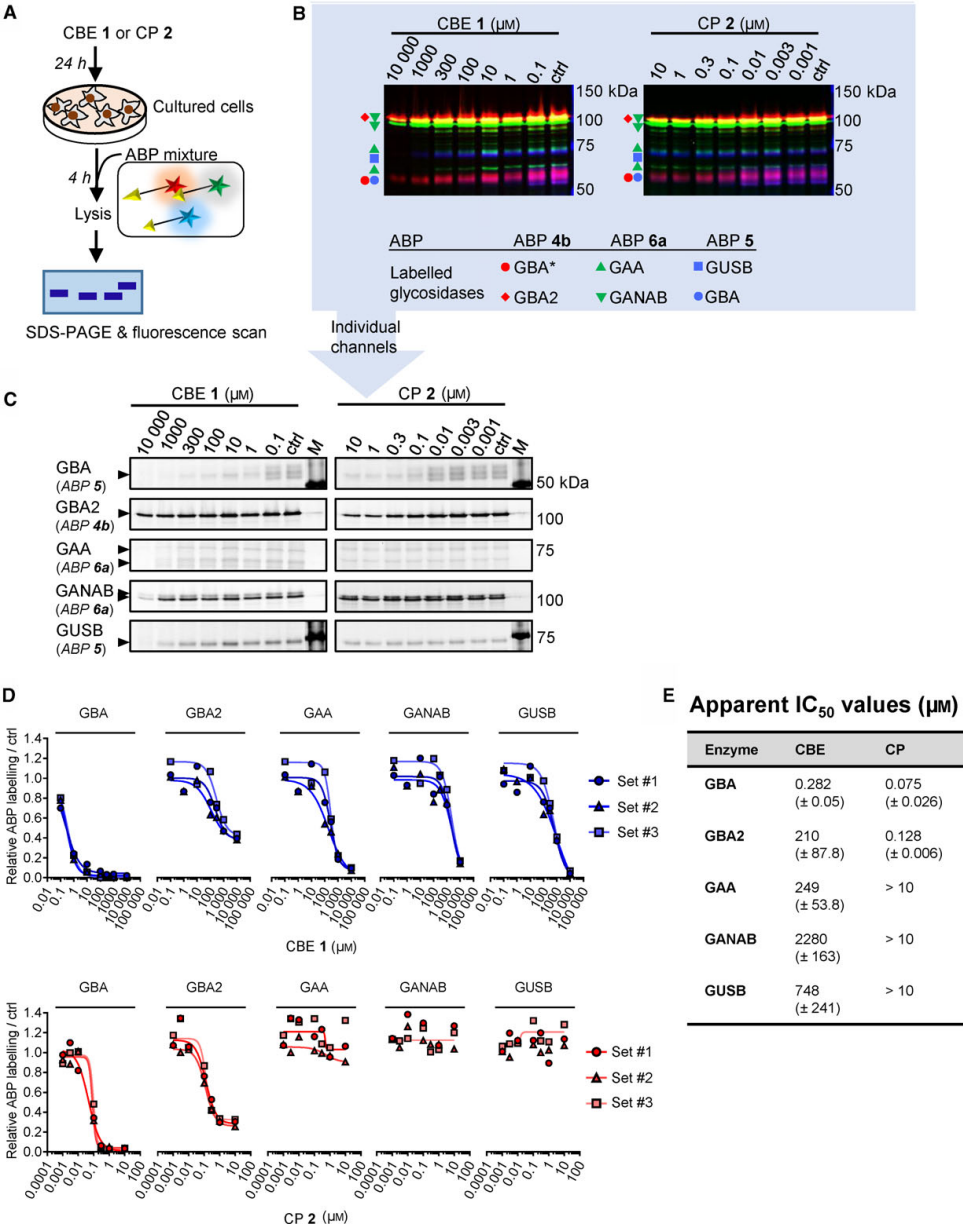
Effect of *in vivo* incubation with CBE and CP on *in vivo* ABP labelling of glycosidases in cultured cells. (A) Schematic representation of the experimental procedure. (B) Overlay of fluorescence channels showing *in vivo* ABP labelling of glycosidases that have not been inactivated by CBE or CP; one representative image is selected from *n *= 3 biological replicates. *GBA labeling by ABP **4b** was not used for analysis, due to the presence of an unidentified off‐target by ABP **4b** in living cells. ABP **5** labels both GUSB and GBA 37. (C) Individual fluorescence channels of (B); one representative image is selected from *n *= 3 biological replicates. (D) Quantification of ABP labelling. (E) Apparent IC_50_ values. Error ranges = ±SD,* n *= 3 (biological replicates).

We next analysed the targets of CBE in intact human cells, zebrafish larvae and brain of treated mice. These biological materials contain besides GBA the candidate off‐target glycosidases: GBA2, α‐glucosidases (GAA and GANAB) and lysosomal β‐glucuronidase GUSB. For each of these enzymes, ABPs have been designed, and enzymatic activity assays with fluorogenic substrates established 36, 37, 38. Of note, ABP **4** allows simultaneous visualization of active GBA (58–66 kDa) and GBA2 (110 kDa) following SDS/PAGE analysis. First, we studied HEK293T cells expressing GBA2. Cells were exposed for 24 h to different concentrations of CBE (0.1 μm–10 mm) after which the residual amount of GBA, GBA2, GAA, GANAB and GUSB in cell lysates that can still be labelled with the appropriate ABPs (Fig. 1C) was determined (Fig. 3A). Competitive ABPP showed that besides GBA, all other candidate off‐target enzymes are inactivated by CBE but with marked lower affinity: GBA (IC_50_ = 0.59 μm), GBA2 (IC_50_ = 315 μm), GAA (IC_50_ = 249 μm), GANAB (IC_50_ = 2900 μm) and GUSB (IC_50_ = 857 μm). (Fig. 3B, Table 2). Comparable results were obtained by determination of residual enzyme activities: GBA (IC_50_ = 0.33 μm), GBA2 (IC_50_ =272 μm), GAA (IC_50_ = 309 μm), GANAB (IC_50_ = 1580 μm) and GUSB (IC_50_ = 607 μm) (Fig. 3C, Table 2). We used cultured human fibroblasts, rich in lysosomal enzymes, to analyse a panel of additional glycosidases (α‐ and β‐mannosidase, *N*‐acetyl α‐galactosidase, β‐hexosaminidase, α‐fucosidase, α‐iduronidase, α‐ and β‐galactosidase) for their possible inactivation by CBE. At the highest concentration of CBE (10 mm) tested, no significant loss of activity was observed for any of these additional lysosomal enzymes (Fig. 4). In addition, we also performed *in vivo* competitive ABPP experiments. For this, the appropriate ABPs were added to the culture medium of HEK293T cells during 4 h prior to their harvesting. The outcome of this *in vivo* ABP labelling was comparable to that of *in vitro* ABP labelling in the lysates of harvested cells (Fig. 5).

**Table 2:** *In vivo i*nhibition of CBE or CP towards glycosidases in cultured cells. Apparent IC_50_ values (μm) were derived from the average of biological triplicates as measured by either *in vitro* enzymatic assays (*Enz. assay*) or quantification of *in vitro* ABP labelling on residual active enzymes (*ABP*). ‐, value cannot be calculated (no inhibition at the tested concentrations). Error ranges = ±SD, *n *= 3 biological replicates

Enzyme	CBE		CP	
	Enz. assay	ABP	Enz. assay	ABP
GBA	0.331 (±0.189)	0.594 (±0.316)	0.086 (±0.003)	0.063 (±0.035)
GBA2	272 (±101)	315 (±62.8)	0.198 (±0.008)	0.154 (±0.070)
GAA	309 (±88.2)	249 (±83.9)	–	–
GANAB	1580 (±116)	2900 (±1120)	–	–
GUSB	607 (±70.0)	857 (±341)	–	–

**Figure 6:**
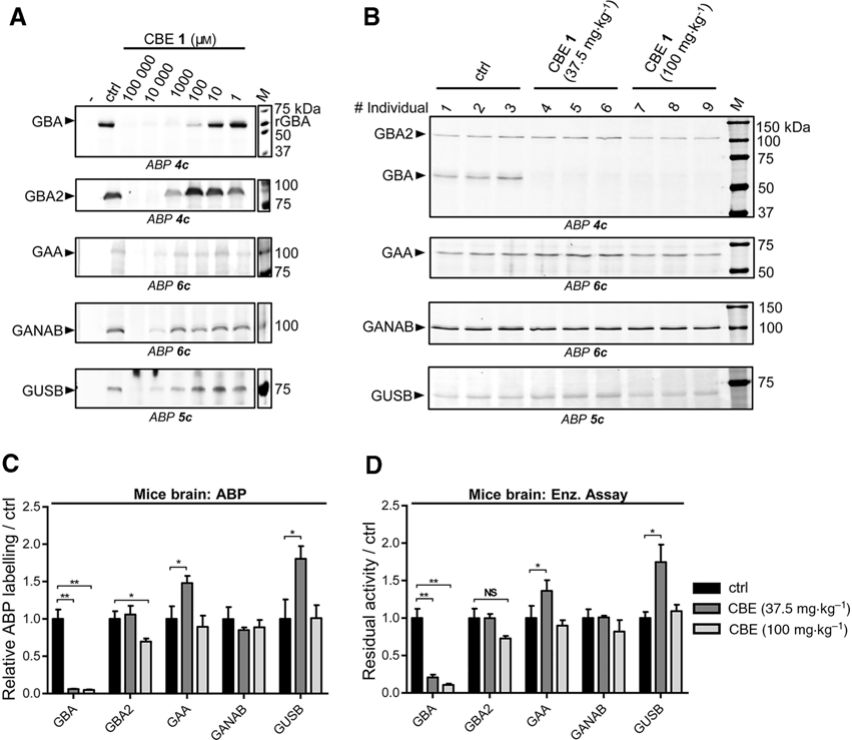
*In vivo* glycosidase targets of CBE in animal models. (A) Effect of CBE incubation in zebrafish to ABP labelling of glycosidases in zebrafish lysates. (B) Effect of CBE injection in mice to ABP labelling of glycosidases in mice brain homogenates. (C) Quantification of labelled bands in (B). (D) Residual activity of glycosidases in brain homogenates of CBE‐injected mice. Error ranges = ±SD,* n *= 3 (biological replicates). Two‐tailed unpaired *t*‐test was used to derive statistical significance (*P < 0.05, **P < 0.01, NS, not significant).

**Figure 7:**
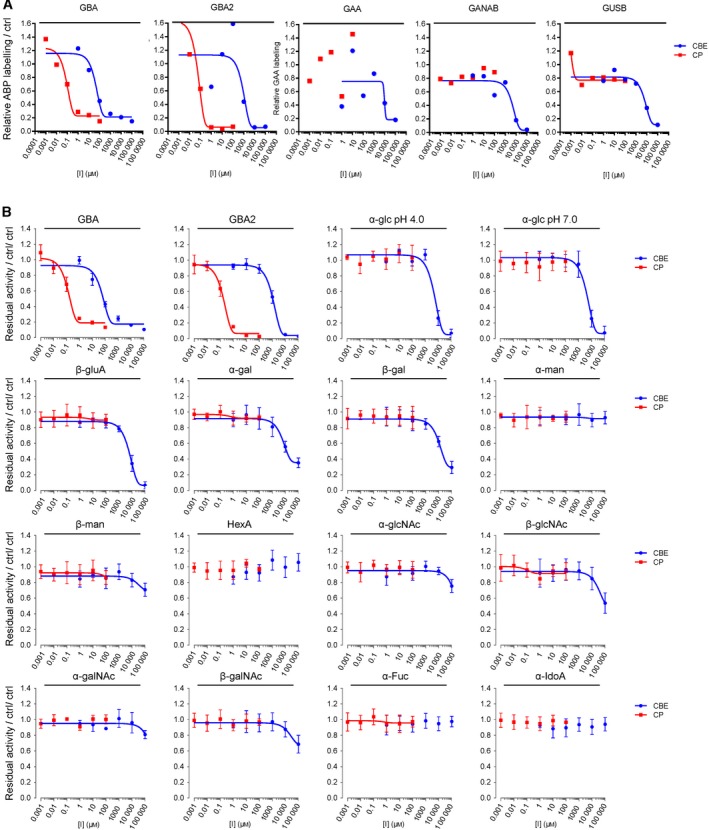
Effect of *in vivo* incubation with CBE and CP on glycosidases in zebrafish larvae. Zebrafish embryos (*n *= 48) were incubated with CBE or CP from 8–120 hpf before lysed in potassium phosphate buffer. (A) Quantification of gels from Figure 6A and 8D. (B) Residual activity of glycosidases by enzymatic assay. Error ranges = ± SD,* n *= 2 technical replicates.

Next, we investigated the CBE target engagement in intact zebrafish larvae. We exposed fertilized zebrafish eggs (0 dpf) to CBE (1 μm–100 mm) in the egg water for 5 days. The larvae were collected, lysed and analysed by the competitive ABPP method. Exposure to 100 mm CBE was found to reduce ABP labelling of all five glycosidases (Figs 6A, 7). The IC_50_ values determined by the competitive ABPP method were: GBA (IC_50_ = 44.1 μm), GBA2 (IC_50_ = 890 μm), GAA (IC_50_ = 9550 μm), GANAB (IC_50_ = 4700 μm) and GUSB (IC_50_ = 6470 μm) (Table 3). Thus, inactivation of GBA in zebrafish larvae takes place 20‐fold more avidly than that of GBA2 and 100–200‐fold more potently than that of GAA, GANAB and GUSB. Analysis of residual enzymatic activity of the various enzymes gave similar results (Table 3, Fig. 7). Analysis by enzymatic assay revealed that exposure of the animals to 10 mm CBE did not lead to significant inactivation of other glycosidases (α‐ and β‐mannosidase, *N*‐acetyl α‐galactosidase, β‐hexosaminidase, α‐fucosidase and α‐iduronidase) except for β‐galactosidase (IC_50_ = 11.2 mm) and α‐galactosidase (IC_50_ = 22.5 mm) (Fig. 7).

**Table 3:** *In vivo* inhibition of CBE or CP towards glycosidases in zebrafish larvae. Apparent IC_50_ values (μm) were derived from measurements from lysates made with *n* = 48 individuals. *Enz. assay*, enzymatic assay; *ABP*, activity‐based probes detection; –, value cannot be calculated (no inhibition at the tested concentrations)

Enzyme	CBE		CP	
	Enz. assay	ABP	Enz. assay	ABP
GBA	58.5	44.1	0.130	0.083
GBA2	1160	890	0.176	0.059
GAA	5010	9550	–	–
GANAB	4550	4700	–	–
GUSB	6380	6470	–	–

Finally, we also investigated the *in vivo* targets of CBE in brain of mice treated daily from day 8 to either day 25 with 37.5 mg CBE·kg^−1^ body weight or to day 14 with 100 mg CBE·kg^−1^ body weight 9. Brain homogenates were prepared from three individuals of each treatment group and the untreated group. As above, both competitive ABPP and measurement of residual enzymatic activity was performed. Interestingly, GBA was found to be the only enzyme significantly targeted by CBE in the brain of mice treated with both CBE dosages (Fig. 6B). Quantification of the gels revealed only a slight reduction in ABP labelling of GBA2 in brain of mice treated with 100 mg CBE·kg^−1^ body weight (Fig. 6C), but this reduction (30%) is far less pronounced than the complete reduction of GBA labelling in the same mice. Measurement of residual enzymatic activities in these samples yielded comparable results (Fig. 6D), showing that besides GBA the other enzymes GBA2, GAA, GANAB and GUSB were not affected in the brains of CBE‐treated mice. Taking together these results, we can confirm that there is a therapeutic window for selective inactivation of GBA with CBE. However, when using higher CBE concentration, looking whether concomitant inhibition of GBA2 takes place is warranted.

### *In vivo* targets of CP

**Figure 8:**
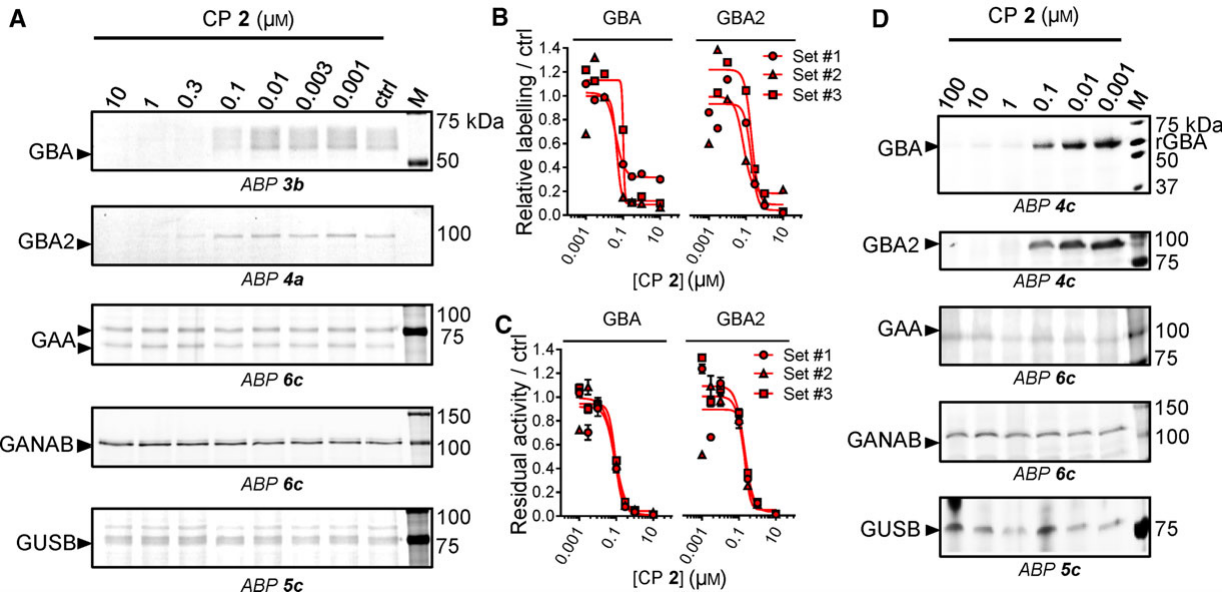
*In vivo* glycosidase targets of CP. (A) Representative gel images (one of three biological replicates) showing fluorescent ABP labelling of GBA, GBA2, GAA, GANAB and GUSB in lysates of cells treated *in vivo* for 24 h with CP. (B) Quantification of relative ABP labelling of GBA and GBA2. (C) Residual activities of GBA and GBA2 in cell lysates treated *in vivo* with CP. Error ranges = ±SD,* n *= 3 (technical replicates). (D) Effect of CP incubation in zebrafish to ABP labelling of glycosidases in zebrafish lysates.

Cyclophellitol has been previously used to generate a GD mouse model 30. Compared to CBE, CP is a much more potent inhibitor of GBA 30 and reported to inhibit poorly α‐glucosidases *in vitro* 31. However, its *in vivo* reactivity towards other glycosidases has not been thoroughly investigated. We therefore comparatively studied the *in vivo* targets of CP in cultured cells and zebrafish larvae using the competitive ABPP methodology and measurement of the residual enzymatic activities. To compare the selectivity windows of CP to the ones of CBE, the concentration range of CP in living models was chosen at 0.001–10 μm for cultured cells and 0.001–100 μm for zebrafish, so that it would match the extent of GBA inhibition by CBE *in vitro* (Fig. 2A, B). As determined by the competitive ABPP method, CP was found to inhibit GBA2 on a par with GBA in Hek293T cells upon incubation with varying inhibitor concentrations (0.1–10 μm) for 24 h. IC_50_ values of CP for blocking ABP labelling were 0.063 μm for GBA and 0.154 μm for GBA2 (Fig. 8A, B, Table 2). No reduction in ABP labelling of GAA, GANAB and GUSB was observed in lysates of cells incubated for 24 h with the highest concentration of CP (10 μm) (Fig. 8A). As determined by enzymatic assay, the apparent IC_50_ values for inactivation were quite comparable, being 0.086 μm for GBA and 0.198 μm for GBA2 (Fig. 8C, Table 2). For the other enzymes the IC_50_ values exceeded at least 10 μm (Fig. 4, Table 2).

Exposure of zebrafish to CP (5 days at 1–100 μm) also comparably competed GBA and GBA2 labelling, but not that of GUSB, GAA and GANAB (Fig. 8D). This finding was again supported by results obtained from measurement of residual enzymatic activities (Table 3, Fig. 7). From the noted lack of selectivity of CP with respect to inactivation of GBA and GBA2, it is obvious that CP does not allow generation of specific GBA deficiency in cell and animal models.

### Inhibitor sensitivity of GBA3

**Figure 9:**
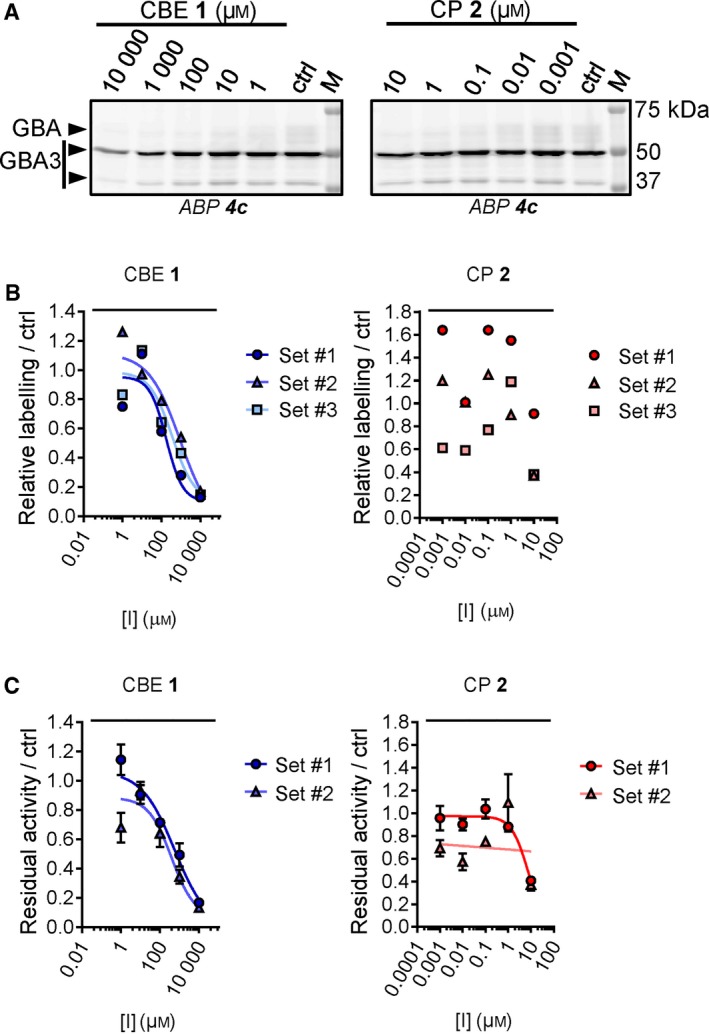
*In vivo* GBA3 inhibition by CBE and CP in cultured cells**.** (A) Representative fluorescent gel images (one of three biological replicates) of ABP labelling of GBA3 in lysates of HEK293T cells treated with CBE or CP for 24 h. (B) Quantification of ABP gels. (C) Residual activities of GBA3 by enzymatic assay. Error ranges = ±SD,* n *= 3 (technical replicates).

Besides GBA and GBA2, there exists in humans and zebrafish another β‐glucosidase named GBA3 (http://www.chem.qmul.ac.uk/iubmb/enzyme/EC3/2/1/21.html) 42. This is a cytosolic enzyme able to degrade a variety of glycoside substrates. GBA3 is not sensitive towards inhibition by CBE 42. The enzyme is selectively expressed in cells and tissues, for example, being particularly high in kidney and absent in fibroblasts, and its activity can be visualized by labelling with nanomolar concentrations of ABP **4** 36. In lysates of mice brain (Fig. 6B) and 5 dpf zebrafish larvae (Figs 6A, 8D), we could not detect the presence of GBA3 (expected m.w. = 45–53 kDa 42) by labelling with 200 nm ABP **4c**. We therefore generated HEK293T cells overexpressing GBA3 to study the inhibitor sensitivity of the enzyme. In these cells we could measure the *in vivo* interaction of CBE and CP with GBA3 by ABP detection (ABP **4c**) (Fig. 9A, B). The apparent IC_50_ values towards GBA3 for CBE and CP were 485 μm and >10 μm, respectively (Table 4). We also measured the inhibition of GBA3 by the two compounds by enzymatic assays, and similar results were obtained (apparent IC_50_ values for CBE and CP were 474 μm and >10 μm; Fig. 9C, Table 4). Hence, both compounds show selectivity towards GBA over GBA3; the ratio of IC_50_ values for GBA3/GBA by ABP detection were 816 for CBE and >159 for CP (Table 4).

**Table 4:** Overview of apparent IC_50_ values (μm) determined in this study. *Enz. assay*, enzymatic assay; *ABP*, activity‐based probes (ABP) detection. Error ranges = ±SD, *n *= 3 biological replicates

Model (incubation time)	Enzyme	CBE		CP	
		Enz. assay	ABP	Enz. assay	ABP
rGBA (3 h)	GBA	4.28 (±0.500)	2.30 (±0.580)	0.030 (±0.002)	0.036 (±0.008)
Cultured cells (24 h)	GBA	0.331 (±0.189)	0.594 (±0.316)	0.086 (±0.003)	0.063 (±0.035)
	GBA2	272 (±101)	315 (±62.8)	0.198 (±0.008)	0.154 (±0.070)
	GAA	309 (±88.2)	249 (±83.9)	>10	>10
	GBA3	474 (±124)	485 (±386)	>10	>10
IC_50_ Ratio	*GBA2/GBA*	*883*	*530*	*2.30*	*2.44*
	*GAA/GBA*	*934*	*418*	*>116*	*>159*
	*GBA3/GBA*	*1430*	*816*	*>116*	*>159*
Danio rerio larvae (5 days)	GBA	58.5	44.1	0.130	0.083
	GBA2	1160	890	0.176	0.059
	GAA	5010	9550	>100	> 100
IC_50_ Ratio	*GBA2/GBA*	*19.8*	*20.2*	*1.35*	*0.710*
	*GAA/GBA*	*85.6*	*226*	*>769*	*>121*

Values and texts marked in italic represent IC_50_ ratio.

### Impact of CBE and CP on glycosphingolipids in exposed zebrafish larvae

**Figure 10:**
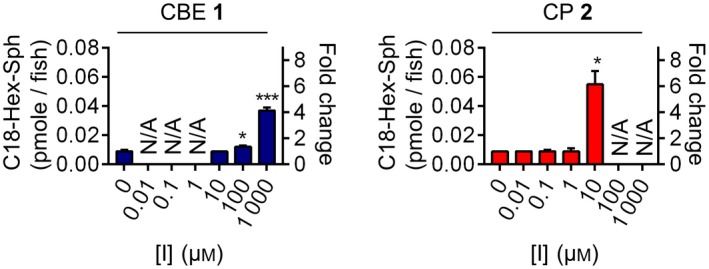
C18‐Hex‐Sphingosine levels in zebrafish larvae treated for 5 days with CBE or CP. Error ranges ± SD,* n *= 3 (biological replicates). Two‐tailed unpaired *t*‐test was used to derive statistical significance (*P < 0.05, ***P < 0.001). *N/A*, not determined.

We next studied the functional impact of exposing fish embryos for 5 days to CBE or CP. It is known that *in vivo* inactivation of GBA leads to compensatory formation of glucosylsphingosine (GlcSph) by acid ceramidase‐mediated conversion of accumulating GlcCer in lysosomes 40, 43. In other words, formation of GlcSph is a biomarker of the inactivation of GBA. The exposure of zebrafish larvae to CBE and CP led to pronounced accumulation of GlcSph (fourfold increase in the case of 1000 μm CBE; sixfold increase at 10 μm CP) (Fig. 10). Thus, the observed *in vivo* GBA inhibition by both compounds according to ABP detection was confirmed by the accumulation of GlcSph at comparable inhibitor concentrations.

## Discussion

Our present study demonstrates that gel‐based fluorescence ABPP can be appropriately used to determine *in vivo* target engagement of irreversible glycosidase inhibitors such as CBE and CP. This method provides important insights on dose‐dependent off‐targets of the tested compounds. The off‐targets may vary among cells and organismal models. From our findings, we conclude that it is wise to test pharmacologically induced Gaucher disease models with respect to selectivity of GBA inactivation.

**Figure 11:**
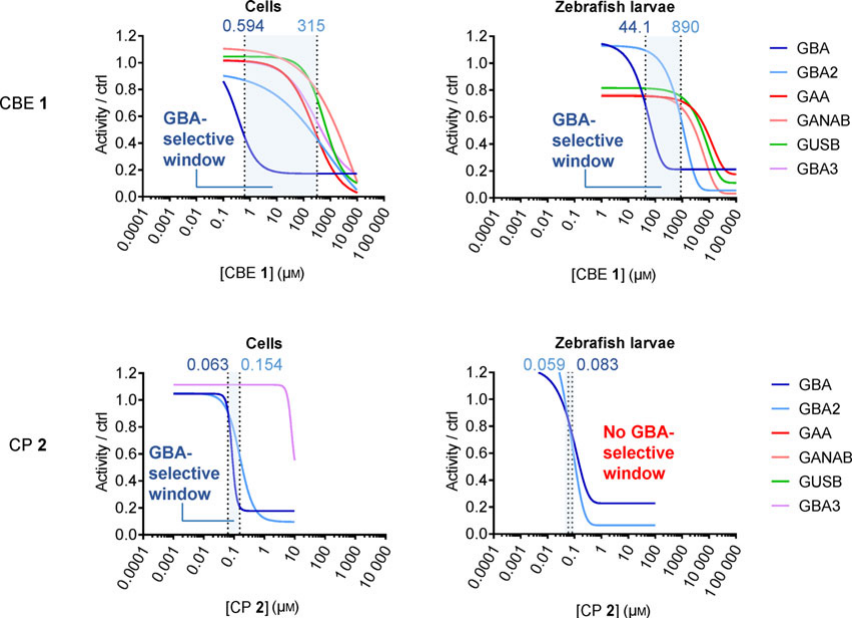
Windows for selective GBA inhibition by CBE and CP in cultured cells and zebrafish larvae. Inhibition curves for CBE or CP towards GBA and other glycosidases were derived from the results of ABP detection (average of *n *= 3 biological replicates). GBA selective window is shown in blue area, defined as the concentration range for CBE or CP between its IC_50_ values towards GBA and the next major glycosidase target (in all cases, GBA2).

We assessed the *in vivo* target engagement of CBE and compared this in cells and zebrafish model to that of CP. Table 4 provides an overview of the IC_50_ values observed for CBE and CP across rGBA, cultured cells and zebrafish larvae. The table highlights that although CBE has *in vivo* off‐targets such as GBA2 and the lysosomal α‐glucosidase GAA, it is still selective towards GBA in cells (GBA2/GBA inhibition ratio = 530; GAA/GBA inhibition ratio = 418; ABP detection) and in zebrafish larvae (GBA2/GBA inhibition ratio = 20.2; GAA/GBA inhibition ratio = 226; ABP detection). Thus, a selective window for *in vivo* GBA inactivation by CBE exists (in cells: 0.6–315 μm; in zebrafish larvae: 44–890 μm; ABP detection) (Fig. 11). Importantly, such selectivity has also been observed by us in brain of mice treated with 37.5 or 100 mg CBE·kg^−1^ body weight (Fig. 6B). It therefore can be concluded that the CBE treatment generates a valuable neuronopathic Gaucher disease model. It should, however, be stressed that the use of higher CBE concentrations or longer incubation periods may cause undesired inhibition of other glycosidases.

Our investigation also revealed that CP is a more potent GBA inactivator. However, CP inhibits GBA2 on a par with GBA in both cultured cells and zebrafish larvae (Table 4), and therefore does not offer a window for selective GBA inactivation (Fig. 11). In other words, CP seems of little use to generate a Gaucher disease model. Previous work showed that a CP functionalized at C6 with a BODIPY moiety is an ABP that potently and selectively inactivates GBA 32. This compound was found not to penetrate well into the brain, likely by active removal via Pgp proteins 39, 44. It will be of interest to design CP analogues that inactivate GBA with higher selectivity and concomitantly are brain permeable. Such compounds might create the desired genuine Gaucher disease models solely deficient in GBA.

## Experimental procedures

### General materials and methods

Cyclophellitol (CP) and the ABPs were synthesized as described earlier 32, 36, 37, 38, 45. Chemicals were obtained from Sigma‐Aldrich (St. Louis, MO, USA) if not otherwise indicated. Conduritol B epoxide (CBE) was purchased from Enzo Life Sciences (Farmingdale, NY, USA). Recombinant GBA (rGBA, imiglucerase) was obtained from Sanofi Genzyme (Cambridge, MA, USA). Human fibroblasts (CC‐2511) were obtained from Cambrex‐Lonza (East Rutherford, NJ, USA). HEK293T (CRL‐3216) and RAW‐264.7 (TIB‐71) cell lines were purchased from ATCC (Manassas, VA, USA). HEK293T cells overexpressing human GBA2 and GBA3 were generated as previously described 46. Cell lines were cultured in HAMF12‐DMEM medium (Sigma‐Aldrich) for fibroblasts or DMEM medium (Sigma‐Aldrich) for HEK293T cell line, supplied with 10% (v/v) FCS, 0.1% (w/v) penicillin/streptomycin and 1% (v/v) Glutamax, under 5% CO_2_ (fibroblasts) or 7% CO_2_ (HEK293T). Zebrafish (*Danio rerio*) were handled and maintained according to standard protocols (zfin.org). Adult zebrafish were housed at a density of 40 per tank, with a cycle of 14 h of light and 10 h of darkness. Adults, embryos and larvae were kept at a constant temperature of 28.5 °C. Embryos and larvae were raised in egg water (60 μg·L^−1^ sea salt, Sera marin). Synchronized wild‐type ABTL zebrafish embryos were acquired after mating of single male and female couples (both > 3 months old). Frozen brain samples from wild‐type and CBE‐treated mice were obtained from a previous study 9. Protein concentration was measured using Pierce BCA assay kit (Thermo Fisher Scientific, Waltham, MA, USA).

### Enzymatic assays

All assays were performed in 96‐well plates at 37 °C for human, zebrafish and mice material. Samples were diluted with McIlvaine buffer to a final volume of 25 μL, at pH appropriate for each enzyme. Assays were performed by incubating the samples with 100 μL 4MU‐ (4‐methylumbelliferyl‐) substrates diluted in McIlvaine buffer (150 mm citric acid—Na_2_HPO_4_, 0.1% (w/v) bovine serum albumin) for a period of 30 min to 2 h. After stopping the substrate reaction with 200 μL 1M NaOH‐glycine (pH 10.3), 4MU‐emitted fluorescence was measured with a fluorimeter LS55 (Perkin Elmer, Waltham, MA, USA) using λ_EX_ 366 nm and λ_EM_ 445 nm 32. Measured activities were subtracted with background values (from samples without enzyme), normalized with the average values from the control samples (no inhibitor) and curve‐fitted to inhibitor concentrations using prism 7.0 (GraphPad Software, San Diego, CA, USA) by the [inhibitor] vs response—various slope (four parameters) method to obtain IC_50_ values. The substrate mixtures used for each enzyme are listed as follows: GBA, 3.75 mm 4MU‐β‐D‐glucopyranoside (Glycosynth, Warrington Cheshire, UK) at pH 5.2, supplemented with 0.2% (w/v) sodium taurocholate and 0.1% (v/v) Triton X‐100, and 25 nm *N*‐(5‐adamantane‐1‐yl‐methoxy‐pentyl)‐deoxynojirimycin (AMP‐DNM), a GBA2‐specific inhibitor 47; GBA2, 3.75 mm 4MU‐β‐D‐glucopyranoside at pH 5.8, with pre‐incubation with 1 μm ABP **3a** for 30 min to specifically inhibit GBA activity; α‐glucosidases, 3 mm 4MU‐α‐D‐glucopyranoside at pH 4.0 (GAA) or at 7.0 (GANAB), GUSB, 2 mm 4MU‐β‐D‐glucuronide at pH 5.0; α‐galactosidases, 2 mm 4MU‐α‐D‐galactopyranoside at pH 4.6; β‐galactosidases, 1 mm 4MU‐β‐D‐galactopyranoside at pH 4.3 with 0.2 M NaCl; α‐mannosidases, 10 mm 4MU‐α‐D‐mannopyranoside at pH 4.0; β‐mannosidases, 2 mm 4MU‐β‐D‐mannopyranoside (Glycosynth) at pH 4.2; β‐hexosaminidase HexA, 5 mm 4MU‐β‐D‐6‐sulpho‐2‐acetamido‐2‐deoxy‐glucopyranoside at pH 4.4; β‐hexosaminidases HexA/B, 5 mm 4MU‐β‐*N*‐acetyl‐glucosaminide at pH 4.5; α‐*N*‐acetyl‐galactosaminidase, 1 mm 4MU‐α‐*N*‐acetyl‐galactosaminide at pH 4.5; α‐L‐fucosidase, 1 mm 4MU‐α‐L‐fucopyranoside at pH 5.0, α‐L‐iduronidase, 2 mm 4MU‐α‐L‐iduronide (Glycosynth) at pH 4.0; GBA3, 3.75 mm 4MU‐β‐D‐glucopyranoside at pH 6.0.

### Fluorescent ABP labelling and detection

Residual active, not irreversibly inhibited glycosidases were labelled with excess fluorescent ABPs in the optimum McIlvaine buffer, if not otherwise stated (see above). ABP labelling was performed at 37 °C for 30 min for all materials, in a total sample volume of 20–40 μL and 0.5–1% DMSO concentration. GBA was labelled with 200 nm ABP **3b** (pH 5.2, 0.1% (v/v) Triton‐100, 0.2% (w/v) sodium taurocholate), or labelled together with GBA2 using 200 nm β‐aziridine ABP **4c** at pH 5.5. GBA2 was labelled with 200 nm β‐aziridine ABP **4a**,** 4b** or **4c**. The α‐glucosidases GAA and GANAB were first preincubated with 200 nm ABP **4a** for 30 min (pH 4.0 for GAA and pH 7.0 for GANAB), followed by labelling with 500 nm ABP **6a** or **6c** at pH 4.0 or 7.0**.** The β‐glucuronidase GUSB was preincubated with 200 nm ABP **4a** for 30 min, followed by labelling with 200 nm β‐aziridine ABP **5c**. After ABP incubation, proteins were denatured by boiling the samples with 5× Laemmli buffer (50% (v/v) 1 M Tris‐HCl, pH 6.8, 50% (v/v) 100% glycerol, 10% (w/v) DTT, 10% (w/v) SDS, 0.01% (w/v) bromophenol blue) for 5 min at 98 °C, and separated by electrophoresis on 7.5% or 10% (w/v) SDS/PAGE gels running continuously at 90 V(32, 36, 37, 38). Wet slab‐gels were scanned on fluorescence using the Typhoon FLA 9500 (GE Healthcare) at λ_EX_ 473 nm and λ_EM_ ≥ 510 nm for green fluorescent ABP **4a** and **6a**; at λ_EX_ 532 nm and λ_EM_ ≥ 575 nm for ABP **3b** and **4b**; and at λ_EX_ 635 nm and λ_EM_ ≥ 665 nm for ABP **4c**,** 5c** and **6c**. ABP‐emitted fluorescence was quantified using imagequant software (GE Healthcare, Chicago, IL, USA) and curve‐fitted using prism 7.0 (GraphPad Software). After fluorescence scanning, SDS/PAGE gels were stained for total protein with Coomassie G250 and scanned on a ChemiDoc MP imager (Bio‐Rad, Hercules, CA, USA).

### *In vitro* effects of inhibitors on rGBA

For IC_50_ measurements using enzymatic assay, 3.16 ng (53 fmole) of rGBA was prepared in 12.5 μL McIlvaine buffer (150 mm, pH 5.2) supplemented with 0.1% (v/v) Triton X‐100, and 0.2% (w/v) sodium taurocholate, and incubated with 12.5 μL of inhibitors (CBE or CP) diluted in McIlvaine buffer at 37 °C for various time periods. Residual activity of rGBA was measured as described in previous section. For assessing the occupancy of active site pocket by the inhibitors using ABP labelling, the same amount of rGBA was prepared in 10 μL of the same McIlvaine buffer, incubated firstly with 2.5 μL inhibitor dilutions prepared in McIlvaine buffer at 37 °C for various time periods, then with 2.5 μL ABP dilutions prepared in McIlvaine buffer; detection of ABP‐labelled rGBA follows the procedures described in the previous section.

### *In vivo* effects of CBE and CP in intact cultured cells

Confluent HEK293T stably expressing human GBA2 were cultured in 12‐well plates in triplicates with(out) CBE (0.01–10 000 μm) or CP (0.001–10 μm) for 24 h at 37 °C. In addition, confluent human fibroblasts were similarly treated in 15‐cm dishes for 2, 24 and 72 h. For lysis, cells were washed three times with PBS, subsequently lysed by scraping in potassium phosphate buffer [K_2_HPO_4_–KH_2_PO_4_, 25 mm, pH 6.5, supplemented with 0.1% (v/v) Triton X‐100 and protease inhibitor cocktail (Roche, Basel, Switzerland)], aliquoted and stored at −80 °C. After determination of the protein concentration, lysates containing equal protein amount (5–20 μg total protein per measurement) were adjusted to 12 μL with potassium phosphate buffer and subjected to residual activity measurements using enzymatic assay (*n* = 3 technical replicates for each biological triplicate at each treatment condition) or ABP detection (*n* = 3 biological replicates). For *in vivo* ABP labelling, HEK293T cells expressing GBA2 were incubated with culture medium containing CBE (0.015–15 000 μm) or CP (0.001–10 μm) for 24 h. Medium was removed and cells were incubated for 4 h with culture medium containing a mixture of 200 nm ABP **4b**, 1 μm ABP **5c** and 500 nm ABP **6c**, or with DMSO only (negative control). Lysates were prepared and measured for protein concentration as described above, and samples containing 60 μg total protein (diluted with potassium phosphate buffer to 15 μL total volume) were subjected to ABP detection (*n* = 3 biological replicates).

### *In vivo* effects of inhibitors in living zebrafish larvae

Adult zebrafish were not sacrificed for this study; all experiments were performed on embryos/larvae before the free‐feeding stage (120 h, that is, 5 days postfertilization) and did not fall under animal experimentation law according to the EU Animal Protection Directive 2010/63/EU. For *in vivo* inhibitor treatment, a single fertilized embryo was seeded in each well of a 96‐well plate, and exposed to 200 μL CBE (1–100 000 μm), or CP (0.001–100 μm) for 120 h at 28.5 °C. Per condition, *n* = 48 embryos were used. At 120 h (5 dpf), larvae were collected, rinsed three times with egg water, fully aspirated, snap‐frozen in liquid nitrogen and stored at −80 °C until homogenization in 200 μL 25 mm potassium phosphate buffer per 48 individual. Lysis was conducted by sonication with a Polytron PT 1300D sonicator (Kinematica, Luzern, Switzerland) on ice at 20% power for 3 s, and repeated three times. Samples containing 20–45 μg total protein were subjected to ABP detection or enzymatic assay.

### *In vivo* effects of CBE in brain of mice

Brain hemispheres were obtained from mice injected daily with CBE at either 37.5 mg or 100 mg·kg^−1^ body weight, or PBS, from day 8 until day of sacrifice (day 24 for the 37.5 mg CBE·kg^−1^ group; day 14 for the 100 mg CBE·kg^−1^ group) as previously described 9. Brain hemispheres were homogenized in 4× volumes of tissue wet weight in 25 mm potassium phosphate buffer (4× volume/wet tissue weight) with 1.0 mm glass beads using a Fastprep‐24 instrument (MP Biomedicals, Santa Ana, CA, USA) set at 6 m·s^−1^ for 20 s, repeated three times, while chilling samples on ice for 2 min between separate runs. Crude lysates were isolated from the glass beads by pipetting into sterile Safe‐Lock Eppendorf tubes. Homogenates were measured for protein concentration, aliquoted and snap‐frozen in liquid nitrogen. Samples containing 50 μg total protein were subjected to ABP detection or enzymatic assay. Two‐tailed unpaired t‐test was performed in prism 7.0 software (GraphPad Software) to derive statistical significance, where *P *< 0.05 was considered significant.

### *In vivo* effects of inhibitors towards GBA3 in cells

HEK293T cells expressing human GBA3 were cultured, treated (triplicate sets) and lysed in an identical setup as described with GBA2‐expressing HEK293T cells. Lysates (12 μg protein) were subjected to ABP detection using 200 nm ABP **4c** at pH 6.0. For enzymatic assay, lysates were separated from GBA2 and GBA by centrifugation (16 000 ***g*** for 10 min at 4 °C) and incubation of the resulting supernatant with 20 μL concanavalin A sepharose beads (Sigma‐Aldrich) in 200 μL of binding buffer (0.1 m Sodium acetate, 0.1 m NaCl, 1 mm MgCL_2_, 1 mm MnCl_2_, 1 mm CaCl_2_, pH 6.0) for 1 h at 4 °C on a rotor. Next, beads were removed from the supernatant by centrifugation. Five microlitres of the supernatant was added with 20 μL McIlvaine buffer (pH 6.0), and subjected to enzymatic assays for GBA3 activity. Measured activity was normalized with the corresponding protein concentration of each sample, and data were processed as earlier described.

### Sphingolipid extraction and analysis by mass spectrometry in zebrafish larvae

Zebrafish embryos at 8 h postfertilization were seeded in 12‐well plates (15 fish/well, 3 mL egg water/well) and treated with CBE (10–1000 μm) or CP (0.01–10 μm)) for 112 h at 28 °C. Thereafter, zebrafish larvae were washed three times with egg water, and collected in clean screw‐cap Eppendorf tubes (three tubes of three larvae per inhibitor concentration). Lipids were extracted and measured according to methods described previously 48. Briefly, after removing of the egg water, 20 μL of ^13^C‐GlcSph 49 from concentration 0.1 pmol·μL^−1^ in methanol (MeOH), 480 μL MeOH and 250 μL CHCl_3_ were added to the sample, stirred, incubated for 30 min at RT, sonicated (5 × 1 min in sonication water bath) and centrifuged for 10 min at 15 700 g. Supernatant was collected in a clean tube, 250 μL CHCl_3_ and 450 μL 100 mm formate buffer (pH 3.2) were added. The sample was stirred and centrifuged, the upper phase was transferred to a clean tube. The lower phase was extracted with 500 μL MeOH and 500 μL formate buffer. The upper phases were pooled and taken to dryness in a vacuum concentrator at 45 °C. The residue was extracted with 700 μL butanol and 700 μL water, stirred and centrifuged. The upper phase (butanol phase) was dried and the residue was dissolved in 100 μL MeOH. Ten microlitres of this sample was injected to the LC‐MS for lipid measurement. Two‐tailed unpaired t‐test was performed in Prism 7.0 software (GraphPad Software) to derive statistical significance, where *P *< 0.05 was considered significant.

## Conflicts of interest

There are no conflicts of interest declared.

## Author contributions

CLK, WWK and IZ performed the experiments; LTL, AV, AHF, AHM and HPS provided essential materials. CLK, WWK, and MM analysed the data. WWK and MM helped to write the manuscript. CLK, JMFGA and MA wrote the manuscript. AHF, HSO and MA revised the manuscript. HSO and JMFGA conceived and designed the study.
